# Assessment of blood perfusion quality in laparoscopic colorectal surgery by means of Machine Learning

**DOI:** 10.1038/s41598-022-16030-8

**Published:** 2022-08-29

**Authors:** Pasquale Arpaia, Umberto Bracale, Francesco Corcione, Egidio De Benedetto, Alessandro Di Bernardo, Vincenzo Di Capua, Luigi Duraccio, Roberto Peltrini, Roberto Prevete

**Affiliations:** 1 University of Naples Federico II - Interdepartmental Research Center in Health Management and Innovation in Healthcare (CIRMIS), Naples, 80131 Italy; 2 Department of Information Technology and Electrical Engineering, University of Naples Federico II, Naples, 80125 Italy; 3 Department of Advanced Biomedical Sciences, University of Naples Federico II, Naples, 80131 Italy; 4 Department of Public Health, University of Naples Federico II, Naples, 80131 Italy; 5 Department of Electronics and Telecommunications, Polytechnic University of Turin, Turin, 10129 Italy

**Keywords:** Gastrointestinal cancer, Colorectal cancer, Information technology

## Abstract

An innovative algorithm to automatically assess blood perfusion quality of the intestinal sector in laparoscopic colorectal surgery is proposed. Traditionally, the uniformity of the brightness in indocyanine green-based fluorescence consists only in a qualitative, empirical evaluation, which heavily relies on the surgeon’s subjective assessment. As such, this leads to assessments that are strongly experience-dependent. To overcome this limitation, the proposed algorithm assesses the level and uniformity of indocyanine green used during laparoscopic surgery. The algorithm adopts a *Feed Forward Neural Network* receiving as input a feature vector based on the histogram of the green band of the input image. It is used to (i) acquire information related to perfusion during laparoscopic colorectal surgery, and (ii) support the surgeon in assessing objectively the outcome of the procedure. In particular, the algorithm provides an output that classifies the perfusion as *adequate* or *inadequate*. The algorithm was validated on videos captured during surgical procedures carried out at the University Hospital *Federico II* in Naples, Italy. The obtained results show a classification accuracy equal to $99.9\%$, with a repeatability of $1.9\%$. Finally, the real-time operation of the proposed algorithm was tested by analyzing the video streaming captured directly from an endoscope available in the OR.

## Introduction

Indocyanine green (ICG) is a molecule developed in the 1950s at Kodak’s R &D laboratories^1^, applied in the field of infrared photography. This molecule is the first substance discovered capable of emitting fluorescence in the near infrared (NIR) spectrum, as it becomes fluorescent when illuminated with infrared light. This substance has negligible toxicity and is quickly disposed of by the body without side effects, except for rare allergic reactions easy to prevent^2^. In 1959, the Food and Drug Administration (FDA) approved its use in clinical settings^3^, and since then, it has been widely used for diagnostic investigations for pathology affecting heart, eyes, liver, and lungs. This substance is injected into the patient’s vein before surgery or near the tumor mass to be removed the day before surgery. The molecule binds to plasma proteins present in the blood, giving its fluorescent properties to the blood, liver, and biliary circulation^4^.

More recently, ICG has been largely used in the surgical field thanks to the introduction of fluorescence detectors, namely optical systems for excitation and detection of the emitted fluorescence. A relevant topic of research is the adoption of ICG to estimate the perfusion quality in laparoscopic surgery^5–10^. This is essential to assess whether the intestine is adequately perfused; this, in fact, serves as an indication of the outcome of the procedure^11–13^. In fact, a perfusion deficiency at the point where an anastomosis is performed increases the risk of anastomotic dehiscence, which consists in a failure to heal the sutures with the consequent appearance of fistulas and tissue perfusion^14^. Therefore, assessing the quality of the perfusion by means of ICG allows the surgeon to promptly intervene while the surgical procedure is ongoing. The most used technique to verify the perfusion of an intestinal segment is to inject ICG into the patient body. This element makes the blood fluorescent with a green tinge if lightened with infrared light. The evaluation of the intensity and the uniformity of this fluorescence allows to assert if the parts are adequately perfused. This technique was successfully used by Boni^14^ to provide information related to perfusion during colorectal surgery, and assist the surgeon in adopting the best strategy in the phases of colorectal anastomosis (stitching of two conical stumps), often necessary in colorectal interventions. Moreover, other applications of ICG relate to the dynamic discrimination of primary colorectal cancer using systemic indocyanine green with NIR endoscopy^15^, intraoperative ureter identification, and lymph node dissection^16^.

Currently, the fluorescence brightness of ICG is evaluated only qualitatively and subjectively by the surgeon, basing on experience. Indeed, at the state of the art, there are no systems or techniques used to quantify it, and to objectively support the surgeons in their assessment. At the state of the art, several attempts to design systems capable to help the surgeons in the assessment of perfusion quality have been made^17–20^. These approaches are mostly based on the diffusion speed of the indocyanine in the tissues. The perfusion of the colorectal segment is estimated by looking at the gradient of the intensity of ICG fluorescence brightness captured by the camera. The output provided by these systems is a heat map highlighting the intestinal portions characterized by a faster increase of ICG fluorescence brightness after the injection. Furthermore, they try to correlate the heat map with the post-surgery result. However, the methods described in these works are not able to automatically assess if the perfusion is good or not in the analyzed area, as they only provide a graphical output that has to be interpreted by the surgeons subjectively.

To overcome this issue, the branch of technology that is becoming increasingly popular is Artificial Intelligence (AI)^21,22^, and in particular Machine Learning (ML). The capillary diffusion of powerful calculators, along with the effort of researchers to develop effective algorithms^23–25^ have contributed to the widespread adoption of this technology in a large variety of application contexts, such as healthcare^26,27^. At the state of art, there are many examples of ML-based approaches used in the medical field, as proof that this technology can help and assist the operators to minimize the risks for the patients and prevent complications. For example, a decision support system based on AI was used by Cahill et al.^28^ in colorectal cancer intra-operative tissue classification. Instead, Park et al.^29^ adopted AI to evaluate the feasibility of AI-based real-time analysis of microperfusion to predict the risk of anastomotic complication in the patient with laparoscopic colorectal cancer surgery. According to Igaki et al.^30^, a first study to use an image-guided navigation system with total mesorectal excision was conducted. Moreover, Sanchez et al.^31^ provided a systematic literature review regarding the use of AI to find colorectal polyps in colonoscopy. Finally, Kitaguchi et al.^32^ used AI to identify laparoscopic surgical videos, in order to facilitate the automation of time-consuming manual processes, such as video analysis, indexing, and video-based skill assessment. Nevertheless, there are still no methods based on AI that automatically assess the quality of perfusion in the analyzed area.

Starting from these considerations, in this paper, a ML-based system to objectively assess if an intestinal sector is adequately perfused after an injection of ICG is proposed. This system is used to (i) acquire information related to perfusion during laparoscopic colorectal surgery, and (ii) objectively support the surgeon in assessing the outcome of the procedure. In particular, the algorithm provides an output that classifies the perfusion as *adequate* or *inadequate*.

From an implementation point of view, the system works on a video extracted from a laparoscopic camera and a Region of Interest (ROI). The ROI is selected by a member of the operating room team (e.g., an assistant surgeon) and contains the area to be assessed. Then, it adopts a set of pre-processing steps to build the input of a Feed Forward Neural Network used to evaluate the quality of perfusion. The precise tuning of the neural network hyper-parameters allows the proposed architecture to have a prediction accuracy high enough to anticipate the possible adoption of the system as a standard routine to be applied during surgery. As a proof-of-concept demonstration, the case study, based on perfusion analysis applied to abdominal laparoscopic surgery at University Hospital *Federico II* in Naples, Italy, is reported. The feasibility of such approach in real time is proven with optimal performance. Thus, the system can represent an effective decision support for both less-experienced surgeons and those at the beginning of the learning curve.

## Materials and methods

The problem addressed in this work can be formally expressed as a two-class classification problem involving frames (from a video streaming) corresponding to an adequately - or inadequately - perfused area. To this purpose, the idea was to develop a system that automatically assesses the quantity of ICG present in the ROI, by computing the histogram of the green band of the acquired frames, then providing an output corresponding to an *adequate* or to an *inadequate* perfusion.

The study was conducted according to the guidelines of the Declaration of Helsinki. Because the study does not include a pharmacological experimentation, using medical devices or patient data, but only the computer analysis of video material (collected during routine clinical practice), approval by the Ethics Committee is not necessary. Each patient signed an informed consent for the surgical procedure and approved the use of their data by third parties.

### System architecture

**Figure 1:**
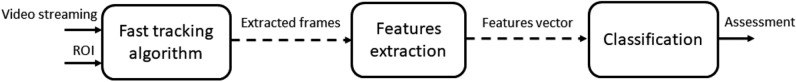
Block architecture of the proposed algorithm. Three main blocks are outlined: (i) a fast tracking algorithm to track the selected ROI, (ii) a feature extraction block to pre-process the available frames and (iii) a ML-based classifier to provide the output in terms of quality of perfusion.

The overall architecture of the proposed system is shown in Fig. 1.

The input of the system are (i) the frames coming from the *Video streaming*, and (ii) the *ROI* identified as a rectangular box selected by the user. The ROI, which identifies the portion of the frame to be analyzed, is selected by the OR operator using the mouse or the track-pad on the computer when starting the algorithm.

The architecture, from left to right, is composed of the following three functional blocks:

- The first block consists of a *Fast tracking algorithm*, which is used to track the selected ROI during the video execution. In particular, the Minimum Output Sum of Squared Error (MOSSE) tracker^33^ was exploited, as it uses adaptive correlation to track objects, resulting in a better robustness to variations in lightning, pose, scale and non rigid transformations. The MOSSE implements also an auto pause and resume functionality if the object to track disappear (for example, if the surgeon covers it) and then it reappears again. Moreover, the exploited tracker can work at high frame rates (more than 450 fps).
- Once the frames containing the ROI are extracted from the video source, the second block performs a *Features extraction*. The frames are in the RGB format, namely the colored image is obtained by a combination of three images, one for each color channel: red, green and blue. Each pixel has an 8-bit resolution; this value represents the intensity of the pixel. Afterwards, the ROI of each frame is divided into 20 vertical equal slices and, for each of them, the histogram of the green band and its area is computed according to Eq. 1:
  1 $$\begin{aligned} A_i= \sum _{l=k}^{255} count_i(l)\cdot [b(l+1)-b(l)]\,\,\,\, 1\le i \le 20 \end{aligned}$$
  where $A_i$ is the generic element of the features vector corresponding to the slice *i*; *b*(*l*) is the bin value at the level *l*; $count_i(l)$ is the number of occurrences of the green intensity at the level *l* for the slice *i*; and *k* is a parameter used to exclude pixels with low values of green. In this work, $k=25$ was chosen as it guaranteed the best classification performance. Finally, a vector of 20 elements (*features vector*) is obtained. This vector becomes the input to the last functional block.
- This step of the process binarily *Classifies* the feature vector and establishes whether it corresponds to an *adequate/1* or to a *inadequate/0* perfusion of the colorectal portion. This classifier is obtained by a Feed Forward Neural Network. The binary cross-entropy was selected as a loss function, and the optimizer *Adam*^34,35^ was chosen.

### Model evaluation and selection

The following Neural Networks (NN) were evaluated as classifiers:

- *One-hidden-layer NN*: In this case, a classic feed forward neural network (FFNN) with one hidden layer was used. The output layer had a single neuron with a sigmoidal activation function. The hidden layer was preliminarly tested with (i) 20 neurons and a *Rectifier Linear Unit* (ReLU) activation function, and (ii) 80 neurons and a *Tanh* activation function. After, this network was further tested with the following activation functions: *Tanh*, *Sigmoid*, and *Rectifier Linear Unit* (ReLU): here, for each activation function the number of neurons changed between 10 and 100, with step 10.
- *Two-hidden-layer NN*: In this case, a FFNN composed by 2 hidden layers was used. Different combinations of *ReLU*, *Sigmoid* and *Tanh* activation function, considering a number of neurons equal to 50, 70, and 90, were tested. *SoftMax* activation function, and two neurons were used for the output layer.

Therefore, the tuned hyper-parameters were (i) activation functions, and (ii) number of neurons for each hidden layer. Moreover, Support Vector Machine (SVM)^36^ method was used with linear and Gaussian kernel as a baseline classifier. For each hyper-parameter configuration, all the aforementioned ML models were validated on the entire data set using the K-fold Cross Validation (CV)^37^ with K=10 folds. K-fold CV is a standard approach to assess and select a ML model in a statistically significant manner and without overfitting^38^. The data set is divided in K folds and the network is trained K times for each combination of hyper-parameters. Each time the network is trained, one of the K folds of the data set is used as test set and all the remaining K-1 as training set. The selection of the best model was conducted according to mean of the obtained accuracies over the K test folds, defined as the percentage of correct classification. After the selection, the chosen model was trained again on the entire data set in order to extract as much information as possible from the data^39^, to use it in real-time in an actual surgery scenario.

The proposed algorithm was developed in *Python 2.7* on Windows 10. The open-source framework and libraries used are *TensorFlow*, *Keras*, and *OpenCV*. The training of the proposed NNs was conducted by setting a number of epochs equal to 100 and the batch size equal to 5.

### Ethical approval

The study was conducted according to the guidelines of the Declaration of Helsinki Approval of the institutional review committee was not required because the data of the present study were collected during routine clinical practice. Each patient signed an informed consent for the surgical procedure and approved the use of their data by third parties.

## Experimental results and discussion

In this section, first, the laboratory experimental validation is described: during this phase, a data set provided by the surgeons was used to train and validate the ML classifiers adopted by the proposed algorithm. Then, an online validation in OR was carried out, by employing the best ML model obtained after the training.

### Laboratory experimental validation

**Figure 2:**
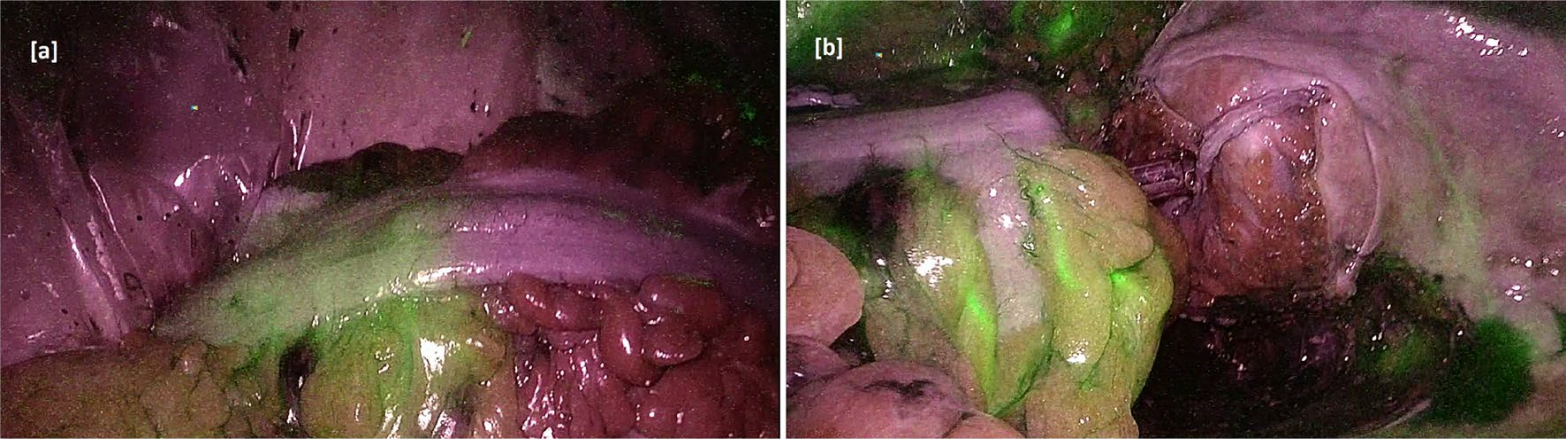
Intraoperative use of ICG technology. Fluorescence angiography shows the vascular perfusion of the intestinal segment that delimits the section point (**a**). Anastomosis is performed with the residual colon (**b**).

**Figure 3:**
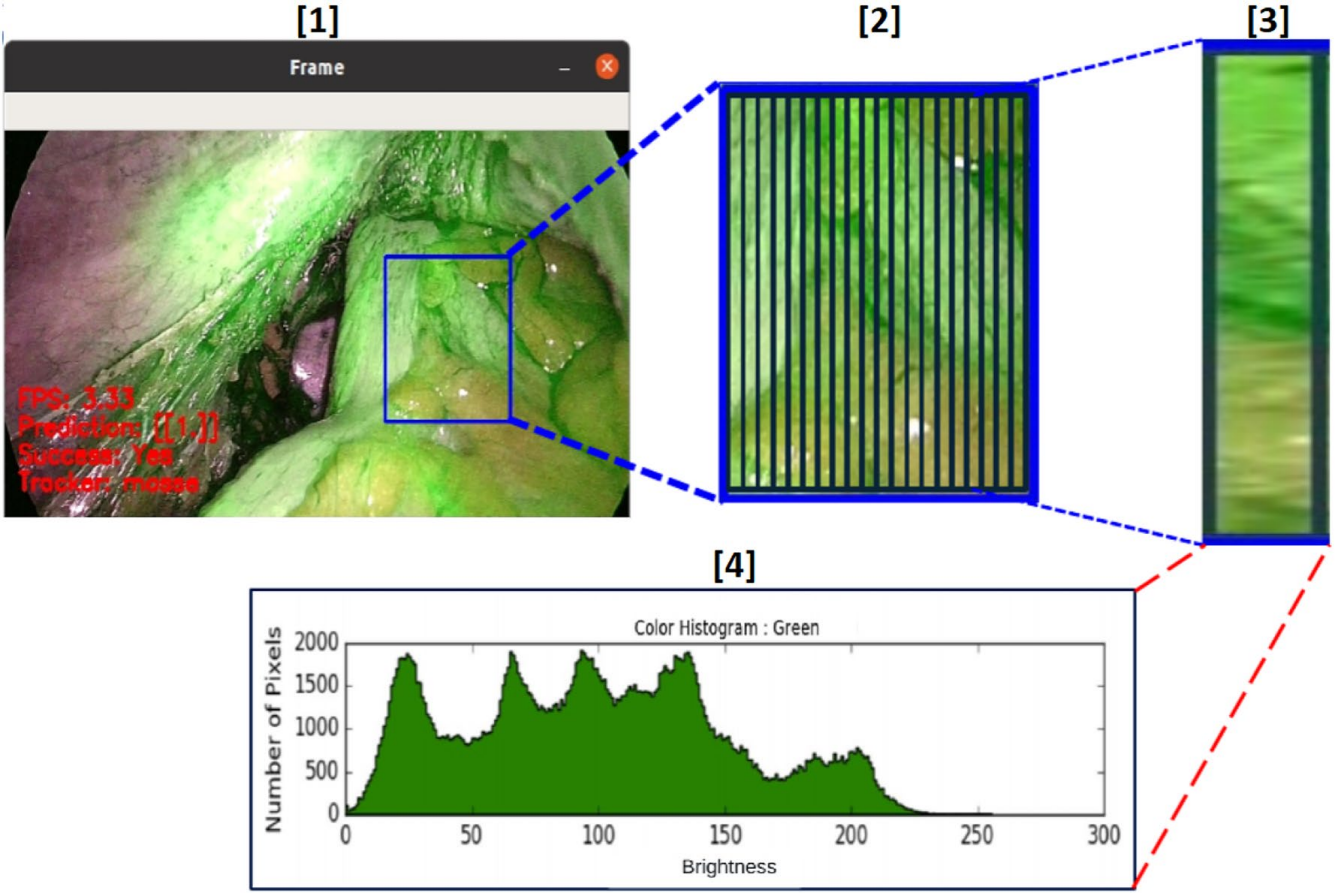
Details about the features extraction: Step 1: selection of the ROI. Step 2: the ROI is divided in 20 slice. Step 3: for each slice, the histogram of the green band is evaluated. Step 4: the amount of green of each histogram is evaluated and the obtained feature is used to build the feature vector sent to the ML classifier.

**Figure 4:**
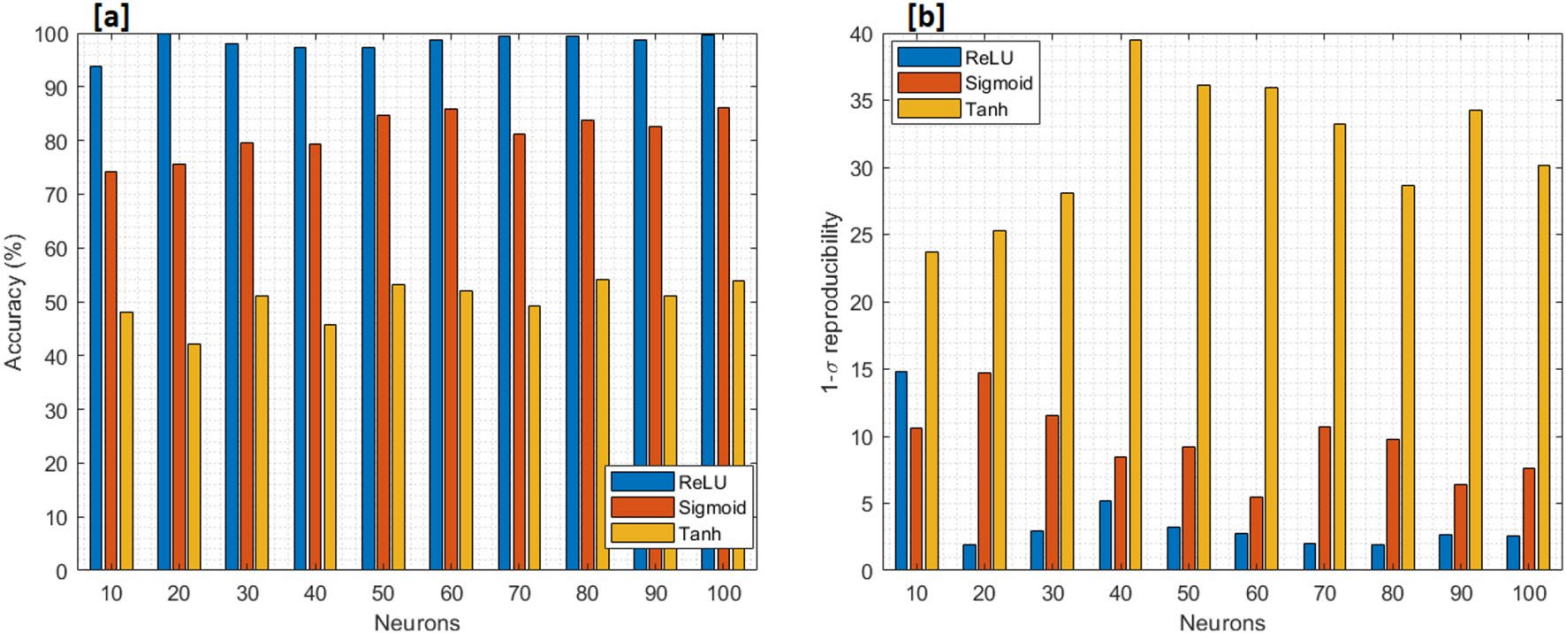
Comparison of (**a**) accuracy, and (**b**) 1-$\sigma$ repeatability for the three activation functions used with different neurons: Tanh (orange), Sigmoid (red), Rectifier Linear Unit (blue).

**Figure 5:**
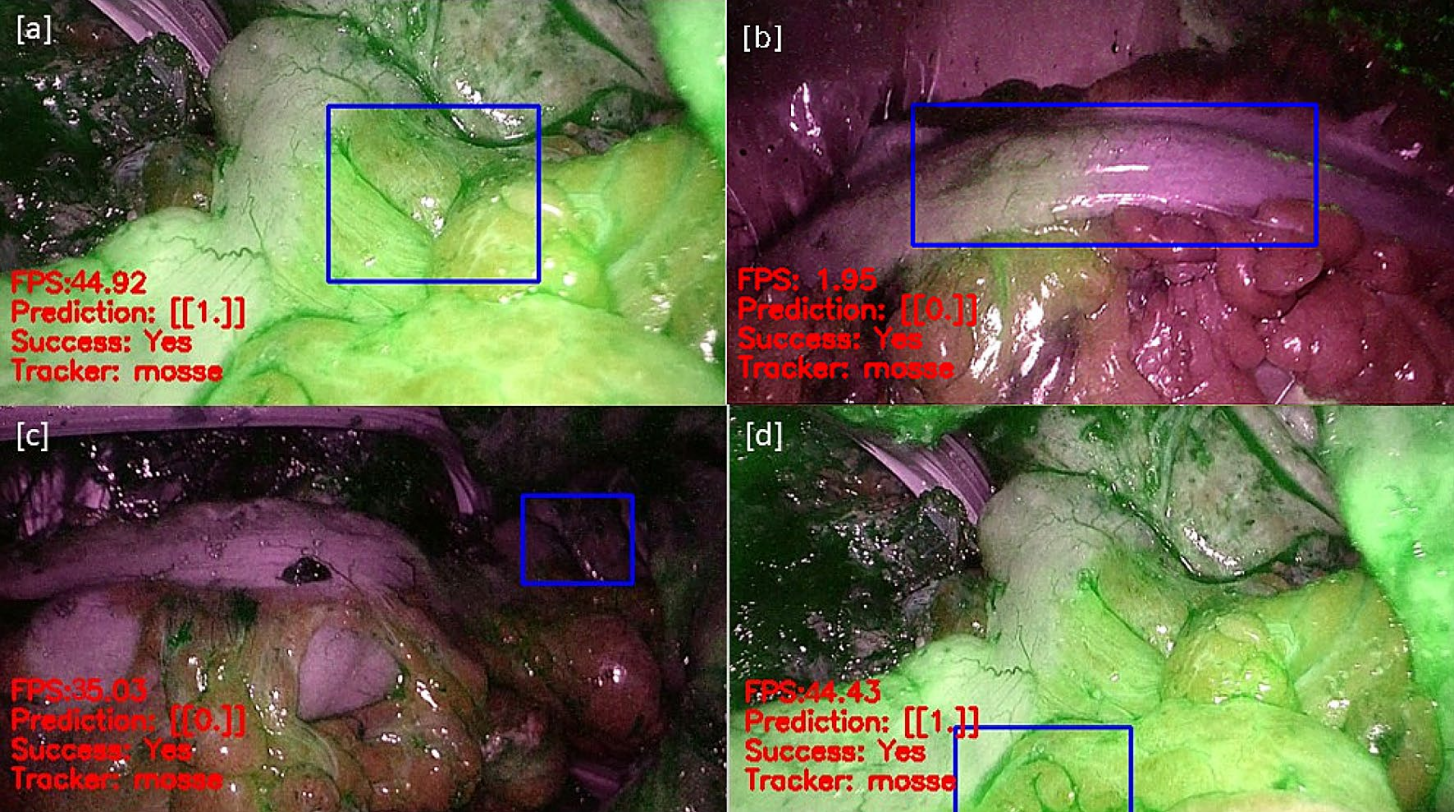
Four frames from the data set with respective ROI: (**a**) and (**d**) have ROI with adequate perfusion (high amount of green) and prediction 1. (**b**) and (**c**) have prediction 0 because the ROIs are inadequately perfused (low amount of green and/or not uniform ICG diffusion).

1. *Setup*
  A total of 11 videos in .M4V format were provided by the surgeons: the videos were collected and labelled by the medical staff during routine clinical practice. An anonymisation procedure was applied to protect patients privacy. In particular, any metadata was removed from the original files. These files contain the video, acquired directly from the endoscope during surgery, related to the portions of the intestine where the anastomosis was being performed. When the ICG was injected, the portion that was well perfused became fluorescent. An example of frames extracted from the dataset is shown in Fig. 2, which shows the intraoperative use of ICG technology.
  In particular, Fig. 2a refers to the fluorescence angiography which shows the vascular perfusion of the intestinal segment that delimits the section point. Fig. 2b shows the anastomosis being performed with the residual colon. Fluorescence angiography was performed using a laparoscopic system (*Olympus OTV-S300*, Olympus Europe SE & Co. KG, Hamburg, Germany) with a light source (*Olympus CLV-S200 IR*) which allowed the use of both visible and near-infrared light.
2. *Results*
  The performance of the developed NNs was validated by processing the 11 videos available from the dataset. From each video of the data set, different frames were extracted. To properly train the model in assessing the quality of perfusion, frames containing ROIs with clear evidence of ICG were selected as well as more tricky ones. From each ROI, 20 features vectors were obtained. The total size of the dataset is constituted by 470 frames. Figure 3 illustrates the overall process of features extraction. The considered frame is shown in Step 1. As aforementioned, the ROI selected by the user is splitted into 20 slices (Step 2); therefore, for each obtained slice (Step 3), the histogram of green, showing the occurrence of each level of green, is computed (Step 4). The area of the histogram constitutes an element of the feature vector. The extracted features are given as input to the aforementioned Classifiers: in Table 1 the chosen set of hyper parameters for each of the classifiers with the corresponding obtained accuracy, in terms of means and $1-\sigma$ repeatability, is summarized.
  The obtained experimental results show that the one-hidden layer (L1) NN with 20 neurons and ReLU as activation function is the one that achieves the best performance, with an average accuracy of $99.9\%$ and a $1-\sigma$ repeatability of $1.9\%$. It outperforms the results obtained with the use of Sigmoidal and Tanh activation functions even with more neurons in the hidden layer. It was also found that both SVM and two-hidden layer NN exhibit worse results than the one-hidden layer networks. In fact, with SVM the best achieved accuracy is $54.5\%$, while with the two-hidden layer NN the best accuracy reached $85.2\%$.
  Since the NN with one hidden layer achieved the best results, further tests were dedicated to fine tune the number of neurons. Table 2 and Fig. 4 summarize the detail of the performance for the one-hidden layer networks as both the number of neurons and the activation function are varied.
  It can be observed that the best results are always obtained using ReLU as activation function. The number of neurons which achieved the greatest accuracy was confirmed to be 20.
  The results reported in Table 2 were statistically validated by means of One-Way *ANOVA* and *Fischer test*, by verifying the statistical significance of the differences between the mean accuracies obtained by the three different activation functions used (Tanh, Sigmoid, and ReLU). The chosen null hypothesis $H_0$ was that the groups belonged to the same population with a significance level $\alpha$ = 1.0%. The test rejected the null hypothesis with a *P*-value = $0.0\%$.
  Therefore, the *Paired t-test* was carried out to understand which of the three groups is different from the others. The significance level $\alpha$ was again set equal to 1.0%. For all three tests the hypothesis $H_0$, that assumed the groups were identical, was rejected. The analysis was conducted by means of the online tool *Statistic Kindgom*^40^. Further details are reported in Table 3.
  The tests confirmed that the results obtained with ReLU activation function and 20 neurons are statistically relevant. In fact, there is a significant difference between this model and the others with different activation functions. Hence, this model was chosen for the classification stage of the proposed system and used in the prototype version.
  For the sake of example, Fig. 5 shows the results obtained by applying the proposed algorithm to frames of the data set with good and bad perfusion.
  For each considered frame, the corresponding ROI is indicated. In particular, Fig. 5a and d have ROI classified with good perfusion (good amount of green) and prediction 1. On the other hand, Fig. 5b and c has prediction output equal to 0 because the ROI are considered by the algorithm as bad perfused due to a low grade of green or due to a not uniform presence of green in the selected ROI. In each of the considered cases, the correctness of the classification was confirmed by the surgeons.

**Table 1:** Performance with the chosen set of hyper parameters for all the tested networks.

Network	Kernel	Neurons L1	Neurons L2	Activation function	Accuracy (%)
SVM	Linear	–	–	–	54.5 ± 15.6
SVM	Gaussian	–	–	–	45.4 ± 23.8
FFNN	–	20	–	ReLU	99.9 ± 1.9
FFNN	–	80	–	Tanh	54.1 ± 28.6
FFNN	–	100	–	Sigmoid	86.0 ± 7.6
FFNN	–	90	90	ReLU	85.2 ± 15.0
FFNN	–	50	50	Tanh	69.9 ± 22.9
FFNN	–	90	70	Sigmoid	68.5 ± 24.2

**Table 2:** Performance of FFNN with one hidden layer and Tanh, Sigmoid, and ReLU as activation functions with different neurons.

Neurons	Tanh accuracy (%)	Sigmoid accuracy (%)	ReLU accuracy (%)
10	47.9 ± 23.7	74.2 ± 10.6	93.7 ± 14.8
20	42.1 ± 25.3	75.6 ± 14.7	99.9 ± 1.9
30	51.1 ± 28.1	79.5 ± 11.5	98.0 ± 3.0
40	45.6 ± 39.5	79.4 ± 8.5	97.4 ± 5.2
50	53.2 ± 36.1	84.6 ± 9.2	97.4 ± 3.2
60	52.1 ± 35.9	85.8 ± 5.5	98.6 ± 2.8
70	49.1 ± 33.2	81.1 ± 10.7	99.3 ± 2.0
80	54.1 ± 28.6	83.7 ± 9.8	99.4 ± 1.9
90	50.9 ± 34.2	82.6 ± 6.4	98.7 ± 2.7
100	53.9 ± 30.2	86.0 ± 7.6	99.7 ± 2.6

**Table 3:** Details about statistical analysis of the three groups.

Test	$H_0$	$\alpha$ (%)	*P*-value (%)	Decision
Fischer test Tanh-Sigmoid-ReLU	Same distribution	1.0	0.0	Reject
t-test Tanh-Sigmoid	Same distribution	1.0	$1.8 {\cdot 10^{-9}}$	Reject
t-test Tanh-ReLU	Same distribution	1.0	$3.5 {\cdot 10^{-9}}$	Reject
t-test ReLU-Sigmoid	Same distribution	1.0	$1.0 {\cdot 10^{-5}}$	Reject

### Operating room experimental validation

**Figure 6:**
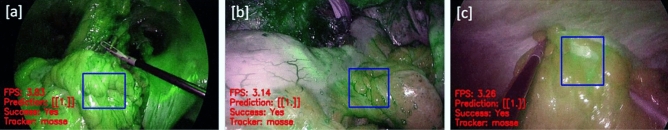
Online validation: Frames characterized by different brightness levels acquired directly from the endoscope during the online validation: (**a**) is characterized by high brightness, (**b**) by medium brightness, and (**c**) by low brightness. Nevertheless, the real-time prediction works even in a low brightness scenario.

1. *Setup*
  After the offline validation, the algorithm was further validated using the equipment available at the University Hospital *Federico II* in Naples, Italy. The aim was to ensure the possibility of interfacing the proposed system with the medical equipment. An additional aspect to consider is, in fact, the real-time interfacing with the endoscope. The endoscope used was the *Olympus Visera Elite II*. It is an imaging platform for general surgery, urology, gynecology, and more, which links the OR to other devices and facilities around the hospital. An S-video to USB adapter was used to connect the endoscope to a PC equipped with Windows 10 and Python 2.7. The video captured from the endoscope was transmitted in real time to an elaboration unit kept outside the OR. Therefore, surgeons who did not take part in the operation were asked to select the ROIs. However, this workflow can be also conducted by the main surgical team inside the OR.
2. *Results*
  The algorithm was able to receive and process at least 30 frame per seconds (fps) from the video source: this frame rate was considered acceptable for the surgeons to select the ROI and use the system. Moreover, the output provided by the algorithm might effectively help surgeons to take the decision even in unclear situations (i.e., low brightness).
  These additional trials demonstrated the feasibility of the practical implementation of the proposed ML-based algorithm Fig. 6, the output of the system working under three different levels of green brightness is shown. This also demonstrates that the proposed system is able to correctly classify the frame regardless of the level of green brightness.

## Conclusions

A system based on ML classifiers is proposed to assist the surgeons during laparoscopic colorectal surgery. It is a decision-support system able to automatically asses if the quality of the perfusion is *adequate* or *inadequate* after an injection of indiocyainine green dye. Different models of classifiers were tested on a dataset of videos of several anastomoses carried out at the Federico II Hospital. Overall, the one-hidden-layer NN with 20 neurons and ReLU activation function achieved the best performance. In fact, the obtained results showed a prediction accuracy of $99.9\%$ with a $1-\sigma$ repeatability of $1.9\%$. These results were statistically validated by means of (i) ANOVA, and (ii) Fischer and Paired-t tests. Therefore, this model was selected for the system implementation.

The proposed system was successfully validated also in relation to the interfacing with actual equipment used at the University Hospital *Federico II* in Naples, Italy. It can represent an important decision support to surgeons during the operation, especially in condition of uncertainty - where it is not clear whether the blood perfusion is adequate or not - due to an unclear presence of ICG.

Future work will be addressed to overcome the current research weakness, by (i) introducing more levels between adequate and inadequate perfusion, in order to increase the resolution of the assessment and further enhance accuracy of prediction, (ii) identifying a method to automatically select the ROIs, and (iii) enrich the dataset by facing circumstances when the blood perfusion is impaired by underlying pathologies (e.g., atherosclerosis). In this case, in fact, both the classifier and the surgeon are not trained to correctly assess whether perfusion is adequate or not.

## Data Availability

The corresponding author is responsible for the safekeeping of all data, available upon request.
